# Impaired ventricular filling limits cardiac reserve during submaximal exercise in people with type 2 diabetes

**DOI:** 10.1186/s12933-017-0644-1

**Published:** 2017-12-19

**Authors:** Genevieve A. Wilson, Gerard T. Wilkins, Jim D. Cotter, Regis R. Lamberts, Sudish Lal, James C. Baldi

**Affiliations:** 1 Department of Medicine, HeartOtago, University of Otago, Dunedin, New Zealand; 2 School of Physical Education, Sports and Exercises Sciences, University of Otago, Dunedin, New Zealand; 3 Department of Physiology, School of Biomedical Sciences, HeartOtago, University of Otago, Dunedin, New Zealand

**Keywords:** Diastole, Systole, Sub-maximal exercise, Echocardiography, Type 2 diabetes, Cardiac reserve

## Abstract

**Background:**

Attenuated increases in ventricular stroke volume during exercise are common in type 2 diabetes and contribute to reduced aerobic capacity. The purpose of this study was to determine whether impaired ventricular filling or reduced systolic ejection were responsible for the attenuated stroke volume reserve in people with uncomplicated type 2 diabetes.

**Methods:**

Peak aerobic capacity and total blood volume were measured in 17 people with diabetes and 16 non-diabetic controls with no evidence of cardiovascular disease. Left ventricular volumes and other systolic and diastolic functional parameters were measured with echocardiography at rest and during semi-recumbent cycle ergometry at 40 and 60% of maximal aerobic power and compared between groups.

**Results:**

People with diabetes had reduced peak aerobic capacity and heart rate reserve, and worked at lower workloads than non-diabetic controls. Cardiac output, stroke volume and ejection fraction were not different at rest, but increased less in people with diabetes during exercise. Left ventricular end systolic volume was not different between groups in any condition but end diastolic volume, although not different at rest, was smaller in people with diabetes during exercise. Total blood volume was not different between the groups, and was only moderately associated with left ventricular volumes.

**Conclusions:**

People with type 2 diabetes exhibit an attenuated increase in stroke volume during exercise attributed to an inability to maintain/increase left ventricular filling volumes at higher heart rates. This study is the first to determine the role of filling in the blunted cardiac reserve in adults with type 2 diabetes.

**Electronic supplementary material:**

The online version of this article (10.1186/s12933-017-0644-1) contains supplementary material, which is available to authorized users.

## Introduction

Maximal exercise capacity is reduced in people with type 2 diabetes (T2D), often reflected by a blunted increase in cardiac output during exercise [1–6]. Heart rate reserve is lower in T2D, because of elevated resting heart rates [7, 8] and reduced [6, 9] or comparable [10–12] peak heart rates. A very consistent finding among T2D is that left ventricular (LV) stroke volume fails to increase, or increases less [3, 13] during exercise. It is unclear however whether the attenuated stroke volume responses are the result of impaired contractility or reduced LV filling.

Diastolic dysfunction, characterised by reduced end-diastolic volumes and impaired early filling (relaxation), is an early manifestation of diabetes that has been implicated as an important contributor to observed reductions in aerobic capacity [8, 14]. However few studies have evaluated LV volumes *during* exercise conditions, i.e., when filling time is reduced and atrio-ventricular pressure gradients are elevated. Indeed, Peterson et al. [15] have shown that peak mitral early to late diastolic filling velocity ratio (E/A), the standard measure of ventricular filling, was inversely proportional to the exercise-induced increase in stroke volume in healthy older subjects, suggesting that early diastolic filling at rest does not predict stroke volumes during exercise, at least in older adults.

Regensteiner et al. [16] evaluated LV performance during submaximal exercise using Swan-Ganz catheters, and found that women with uncomplicated T2D had a more rapid increase in end-diastolic filling pressure than non-diabetic controls. They suggested these findings were consistent with a diastolic limitation to cardiac reserve, however LV volumes were not measured. Pinto et al. [3] reported a smaller increase in stroke volume during submaximal exercise in adolescents with T2D that was reflected by both a smaller LV end-diastolic volume and a smaller decrease in end-systolic volume during exercise. They concluded that, in addition to impaired LV filling, reduced contractility limited cardiac reserve during exercise. However, these measurements were made supine, which does not reflect the physiological LV loading characteristics during *upright* posture [17]. In addition, their study was conducted in adolescents with T2D, whose left ventricular function may differ from adults [18]. Consequently, it is still unclear whether *resting* diastolic impairment, which is common in people with uncomplicated diabetes, limits left ventricular filling and/or impairs contractility during upright exercise.

Therefore, this study aimed to determine the contributions of LV filling and contractility to the LV exercise response of adults with uncomplicated T2D. To achieve this, echocardiographic measurement of LV volumes at semi-recumbent rest and during cycling exercise at 40 and 60% of maximal aerobic capacity were compared in adults with T2D vs. age-, sex- and body composition-matched non-diabetic participants. In addition, total blood volume was quantified to establish what association, if any, this had to left ventricular volumes. We hypothesised that resting cardiac output would be the same between people with and without T2D, but the increase in cardiac output with exercise would be reduced in T2D. We also hypothesized that end-diastolic volume and stroke volume would increase less during exercise in T2D, but LV contractility would not be different.

## Materials and methods

### Participants

Seventeen people with type 2 diabetes (T2D) and 16 non-diabetic controls (CON) between the ages of 34–63 participated in this study. Participants were NZ European (13 T2D, 13 CON), Indian (3 T2D) and NZ Māori (1 T2D, 3 CON). Two participants were current smokers (1 T2D, 1 CON) and 11 were ex-smokers (5 T2D, 6 CON). Participants had no evidence of cardiovascular or respiratory disease and none were taking cardiovascular medications. Four people with diabetes had evidence of mild microvascular disease as assessed by medical records [neuropathy (2), retinopathy (2)]. Participants with diabetes were treated with metformin (n = 14), gliclazide (n = 4) and insulin (n = 3). Control participants were screened identically to participants with diabetes; two were taking statins but were otherwise healthy. Informed consent was obtained from all participants before any testing was undertaken.

### Overview of protocol

Each participant underwent a peak exercise test with electrocardiogram (ECG) to determine $\dot{\text{{V}}}{\text{O}}_{{\text{2peak}}},$ a body composition assessment (DEXA scan), exercise stress echocardiogram and blood volume assessment [19]. Exercise ECGs were analysed by a cardiologist to exclude participants with evidence of cardiovascular disease based on ST segment changes or exercise-induced arrhythmias. No participants had evidence of mitral regurgitation.

### Peak exercise test

Peak oxygen consumption $\left(\dot{\text{{V}}}{\text{O}}_{{\text{2peak}}}\right)$ was determined using a metabolic cart (COSMED Quark CPET, Italy) and an upright cycle ergometer. The exercise protocol began at 25- or 50-W and consisted of 2 min stages with increasing workloads of 25- or 50-W (depending on the participant’s ability) until the termination of the test due to volitional exhaustion. Workloads were adjusted using the Borg scale of perceived exertion to assure test duration did not exceed 12 min. Arm blood pressure (by auscultation) and heart rate (HR, by 12-lead electrocardiogram) were recorded at rest, during each stage of exercise and during recovery. Breath by breath data were collected and analysed using O_2_ and CO_2_ analysers, which were calibrated with room air and standardised gas containing 15% O_2_ and 6% CO_2_. The gas turbine was calibrated by using a 3-L syringe to validate flow rates. The two highest consecutive 10-s recordings were averaged to determine $\dot{\text{{V}}}{\text{O}}_{{\text{2peak}}}.$ Peak HR was not considered a reliable index of peak exercise effort, as it is variable in T2D cohorts [6, 11, 12]. The exercising ECG was assessed by a trained cardiologist before the participant continued in the study to specifically exclude those with evidence of coronary artery disease. At the termination of the test the participant lay immediately in the supine position and heart rate was recorded at 1 and 2 min into recovery for analysis of heart rate recoveries.

### Exercise stress protocol

End diastolic and end-systolic ventricular volumes were determined between 2 and 7 days following the $\dot{\text{{V}}}{\text{O}}_{{\text{2peak}}}$ test. LV volumes (end-diastolic volume, end-systolic volume) were determined. Images were first obtained in a supine position for baseline comparisons between T2D and CON. LV measurements were then repeated in a semi-recumbent position at rest and compared to images obtained while participants pedalled at workloads to achieve heart rates that elicited 40 and 60% of their $\dot{\text{{V}}}{\text{O}}_{{\text{2peak}}}.$ Target HRs were calculated by fitting a binomial equation plotting HR against $\dot{\text{{V}}}{\text{O}}_{{\text{2}}}$ as a percentage of peak. Workloads were adjusted to achieve HR correlating to 40 and 60% of $\dot{\text{{V}}}{\text{O}}_{{\text{2peak}}}$ and echocardiographic images were taken when HR stabilised (∆ < 5 beats min^−1^ of target). HR was recorded throughout the echocardiogram using a single lead ECG and blood pressure was obtained at the end of each stage.

### Echocardiography

Echocardiographic images were obtained using a Vivid E9 (GE Medical systems, Milwaukee, WI, USA) ultrasound system by one trained research sonographer. LV volumes at end-diastole and end-systole (LVEDV, LVESV) were obtained in the apical four-chamber view using two-dimensional echocardiography in accordance with the American Society of Echocardiography (ASE) guidelines [20]. At rest, LV dimensions were obtained in the parasternal long axis view [20] and diastolic filling velocities (E and A) were obtained in the apical four chamber view using pulsed wave Doppler with the sample volume placed between the mitral valve leaflets [21]. Doppler imaging was used to determine diastolic and systolic durations where the diastolic duration was determined as the time from ESV (closure of the aortic valve) to the end of EDV (closure of the mitral valve) and systolic duration was the time from EDV to ESV. Tissue velocities (E′, A′ and S′) were obtained in the apical four-chamber view using Tissue Doppler Imaging [22] with the sample volume placed on the mitral valve annulus at the junction with the inter-ventricular septum. Three consecutive beats were stored digitally and analysed with customized dedicated research software (EchoPAC Version.112.0.0 Advanced Analysis Technologies GE Medical Systems). All analysis was performed blinded by the same research sonographer who carried out the echocardiographic measures. LV EDV and ESV were visually traced with papillary muscles excluded in accordance with ASE guidelines [20] from which stroke volume (EDV − ESV) and ejection fraction (EF: SV/EDV) were derived. Apical-two chamber views could not be obtained in all individuals during exercise and therefore apical four-chamber views were used to estimate volumes. In our laboratory, apical four-chamber images during supine rest showed good agreement (R^2^ = 0.92) when regressed to biplane images (ASE recommendation), therefore we believe the apical four-chamber images provide a valid volume measurement. Volumes were indexed to participants’ fat-free mass obtained from dual-energy X-ray absorptiometry (DEXA scan) because this has been identified as an appropriate scaling method to control for differences in adiposity [23]. Cardiac output was calculated as the average HR during the image acquisition × corresponding stroke volume. Stroke work was calculated as stroke volume (EDV − ESV) × mean arterial blood pressure (MABP) × 0.0136 [24], to compare the work of the left ventricle between T2D and CON. Prior to beginning the study, LV volume measurements were conducted by the same trained sonographer with coefficients of variability ranging from 5.5 to 10.2% across all testing conditions.

### Blood volume assessment

Total haemoglobin mass was measured using carbon monoxide (CO) rebreathing [19]. In brief, the participant rested for 20 min, after which mixed-venous concentrations of haemoglobin ([tHb]) and carboxyhaemoglobin ([HbCO]) were measured in at least triplicate samples obtained from a fingerprick (OSMS, Radiometer, Denmark). Any CO in the participant’s system prior to the protocol was determined by the participant exhaling till residual volume onto a CO meter while wearing nose pegs, and was factored into the final estimate. CO dosage was calculated as body mass × 0.7 mL kg^−1^ (females) and 0.8 mL kg^−1^ (males). However, in cases where BMI > 25 kg m^2^, excessive dosage was avoided by applying a hypothetical body mass to attain a BMI of 25, and dosing at hypothetical body mass × 1 mL kg^−1^. The participant inspired the CO mixed with 100% O_2_, and rebreathed for 2 min at normal tidal volume on a closed system with CO_2_ scrubbing. At 2 min, the participant exhaled until residual volume into the closed system and CO concentration of exhaled gas was measured. At 4 min, exhaled CO was again measured and at 6 min [tHB] and [HbCO] were again measured in at least triplicate, from which Hb mass and thus blood volume were derived.

### Statistical analysis

Baseline group comparisons, peak exercise response and blood volume estimates were compared between T2D and CON using an independent Student T test. Haemodynamic responses to exercise over time were compared using linear mixed models for repeated measures with post hoc tests (LSD and SIDAK) where appropriate. Significance for all two tailed tests was defined as P < 0.05 All data are expressed as mean ± standard error. Analyses were performed using SPSS statistical software (SPSS version 22.0.0, IDM Corporation, Armonk, New York).

## Results

As shown in Table 1 T2D and CON were comparable for age (P = 0.58), BMI (P = 0.17), weight (0.51), fat free mass (FFM) (P = 0.68), body fat % (P = 0.25) and self- reported fitness history (P = 0.54). The mean duration of diabetes among T2D was 8.3 years (ranged 0.6–15 years). As expected, mean [HbA1c] was higher in T2D. Mild microalbuminuria was present in 1 T2D, but there were no other indications of microvascular or autonomic dysfunction in any participants. Blood volume was 11% lower in T2D, however this was not statistically significant (P = 0.09).

**Table 1:** Baseline demographic variables in people with (T2D) and without type 2 diabetes (T2D)

Variable	T2D (n = 17)	CON (n = 16)	*P* value
Age (years)	51 ± 2	52 ± 2	0.58
Sex (M:F)	(8:9)	(8:8)	N/A
Weight [25]	97 ± 6	93 ± 3	0.51
BMI (kg m^2^)	34 ± 2	31 ± 1	0.17
FFM [25]	58 ± 3	60 ± 3	0.68
Body fat (%)	39 ± 72	35 ± 3	0.25
Self reported fitness history (h week^−1^)	3.7 ± 1.0	4.3 ± 0.7	0.54
Diabetes duration (years)	8.3 ± 2.3	N/A	N/A
HbA_1c_ (mmol mol^−1^)	58 ± 3	37 ± 1	< 0.01
Blood volume (mL kg^−1^)	55 ± 3	62 ± 3	0.09

Values are expressed as means ± standard error

*BMI* body mass index

Resting HR was 18% higher in T2D (P = 0.01) however peak HR was not different between groups (P = 0.50). HR reserve was on average 14 beats min^−1^ lower in T2D (P = 0.02) and $\dot{\text{{V}}}{\text{O}}_{{\text{2peak}}}$ was approximately 10% lower (P = 0.04) (Table 2). Maximal workload was reduced in T2D (P = 0.01) while exercise duration was not different (P = 0.23). HR recovery was not different at one or 2 minutes after ceasing exercise (P = 0.57 and P = 0.13 respectively). All but one participant (T2D) achieved an RER (i.e. CO_2_/O_2_) > 1.10.

**Table 2:** Exercising and blood volume variables in people with (T2D) and without type 2 diabetes (T2D)

Variable	T2D (n = 17)	CON (n = 16)	P value
Resting HR (beats min^−1^)	71 ± 3	60 ± 2	0.01
Peak HR (beats min^−1^)	165 ± 4	168 ± 4	0.50
HR reserve (beats min^−1^)	94 ± 4	108 ± 4	0.02
$\dot{\text{{V}}}{\text{O}}_{{\text{2peak}}}$ (mL kg^−1^ min^−1^)	22 ± 2	26 ± 2	0.06
˙$\dot{\text{{V}}}{\text{O}}_{{\text{2peak}}}$ (mL kg_FFM_^−1^ min^−1^)	36 ± 2	40 ± 2	0.04
Max workload (W)	144 ± 14	205 ± 19	0.01
Exercise test duration (min)	7.7 ± 0.5	8.6 ± 1.9	0.23
HR recovery 1 min (beats min^−1^)	31 ± 3	30 ± 2	0.57
HR recovery 2 min (beats min^−1^)	50 ± 3	56 ± 2	0.13

Values are expressed as means ± standard error

*HR* heart rate

There were no differences in LV mass (absolute and indexed to BSA) supine resting Q̇_FFM_, SV_FFM_, EF, EDV_FFM_, ESV_FFM_, TPR and MABP. Stroke work was 18% lower in T2D (P = 0.03). The E/A ratio was lower and A was greater in T2D (P < 0.01, P < 0.01 respectively) while deceleration time, E and E/E′ were not different (P = 0.43, P = 0.79, P = 0.11 respectively) (Table 3). E′ was reduced in T2D (P = 0.01) however A′ and S′ were not different between the groups (P = 0.93, P = 0.40 respectively). T2D spent 60% of their resting cardiac cycle in diastole while CON spent 65% although this was not significant (P = 0.06).

**Table 3:** Left ventricular echocardiographic variables at rest in the supine position

Variable	T2D (n = 17)	CON (n = 16)	P value
HR (beats min^−1^)	73 ± 3	62 ± 2	< 0.01
LV mass (g)	209 ± 14	192 ± 9	0.30
LVMI (g m^−2^)	101 ± 5	92 ± 3	0.15
Q̇_FFM_ (L min^−1^ kg_FFM_^−1^)	0.08 ± 0.00	0.08 ± 0.00	0.40
SV_FFM_ (mL kg_FFM_^−1^)	1.17 ± 0.06	1.31 ± 0.06	0.12
EF (%)	59 ± 2	61 ± 2	0.59
EDV_FFM_ (mL kg_FFM_^−1^)	1.98 ± 0.07	2.16 ± 0.08	0.11
ESV_FFM_ (mL kg_FFM_^−1^)	0.80 ± 0.04	0.84 ± 0.05	0.48
TPR (mmHg min L^−1^)	21 ± 1	20 ± 1	0.68
MABP (mmHg)	97 ± 3	98 ± 2	0.97
SW (mL mmHg^−1^)	88 ± 6	107 ± 5	0.03
E (cm s^−1^)	66 ± 4	67 ± 3	0.79
A (cm s^−1^)	70 ± 3	55 ± 2	< 0.01
E/A	0.96 ± 0.06	1.25 ± 0.06	< 0.01
Deceleration time (ms)	204 ± 8	194 ± 11	0.43
E′ (cm s^−1^)	7 ± 0	9 ± 1	0.01
A′ (cm s^−1^)	9 ± 1	9 ± 0	0.93
E′/A′	0.85 ± 0.06	1.10 ± 0.08	0.01
S′ (cm s^−1^)	7 ± 0	8 ± 0	0.40
E/E′	10.2 ± 1.1	7.9 ± 0.8	0.11
Time spent in diastole (%)	60 ± 1	65 ± 2	0.06

Values are presented as mean ± SE

*LVMI* left ventricular mass index, *HR* heart rate, *FFM* fat free mass, *Q̇* cardiac output, *SV* stroke volume, *EF* ejection fraction, *EDV* end*-*diastolic volume, *ESV* end*-*systolic volume, *TPR* total peripheral resistance, *MABP* mean arterial blood pressure, *SW* stroke work

**Fig. 1:**
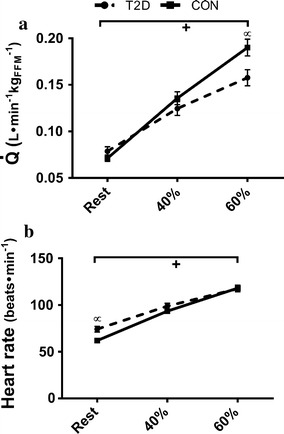
Cardiac output ($\dot{\text{{Q}}},$ **a**) and heart rate (**b**) responses from rest to 40 and 60% of maximal aerobic capacity. ^+^P < 0.05 significant difference across conditions. ^∝^P < 0.05 significant difference between T2D and CON at given intensity. Values are presented as mean ± SE

**Fig. 2:**
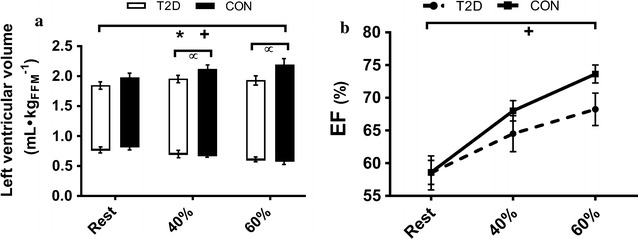
**a** Left ventricular volume responses from rest to 40 and 60% of maximal aerobic capacity. The top of each bar represents mean end-diastolic volume, the bottom represents mean end-systolic volume and the difference (top–bottom) represents the stroke volume. **b** Ejection fraction (EF) response from rest to 40 and 60% of maximal aerobic capacity. *P < 0.05 significant difference between T2D and CON. ^+^P < 0.05 significant difference across conditions. ^+^P < 0.05 significant difference between T2D and CON at given intensity. Values are presented as mean ± SE

**Fig. 3:**
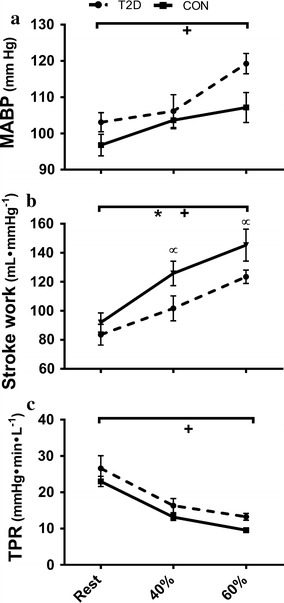
Top: mean arterial blood pressure (MABP, **a**), middle: stroke work (**b**) and bottom: total peripheral resistance (TPR, **c**) responses from rest to 40 and 60% of maximal aerobic capacity. *P < 0.05 significant difference between T2D and CON. ^+^P < 0.05 significant difference across conditions. ^∝^P < 0.05 significant difference between T2D and CON at given intensity. Values are presented as mean ± SE

HR increased in both groups during steady state exercise at 40 and 60% of $\dot{\text{{V}}}{\text{O}}_{{\text{2peak}}},$ but the increase was less among T2D (P = 0.02) due to an elevated resting HR (P = 0.01) (Figs. 1 and 2, see Additional file 1: Table S1 for values). Resultant HRs were not different between the groups at either workload (P = 0.20 and P = 0.88 at 40 and 60% of $\dot{\text{{V}}}{\text{O}}_{{\text{2peak}}}$). EDV_FFM_ was lower in T2D at rest and during both exercise stages (P = 0.01) but the change in EDV_FFM_ during exercise was not different between groups (P = 0.25) (Fig. 2). ESV_FFM_ was not different between groups (P = 0.80), and decreased during exercise in both groups (P < 0.01). The reduction in ESV_FFM_ was not different between groups 1 at either exercise intensity (P = 0.36) (Fig. 2). SV_FFM_ was lower in T2D (P = 0.05) and increased less with increasing exercise intensity (interaction P < 0.01) (Fig. 2). EF (Fig. 3) and Q̇_FFM_ (Fig. 1) were not different between the groups (P = 0.34, P = 0.17 respectively) but increased less in T2D with increased exercise intensity (interaction; P = 0.03, P = 0.01 respectively). MABP and TPR were not different between the groups (P = 0.84, P = 0.08) and increased similarly in both groups as exercise intensity increased (P < 0.01, P < 0.01) (Fig. 3). Stroke work was reduced in T2D (P = 0.02) and increased less during exercise T2D (P = 0.02) (Fig. 3). Peak mitral annular velocity during systole (E′) increased during exercise (P < 0.01) in both groups, but there were no differences between groups (P = 0.17). Peak mitral annular velocity during early relaxation (E′) increased in both groups during exercise (P < 0.01) and was lower in T2D at rest and 40% of $\dot{\text{{V}}}{\text{O}}_{{\text{2peak}}}$ (P = 0.01), however there was no interaction. Measurements were not collected at 60% of $\dot{\text{{V}}}{\text{O}}_{{\text{2peak}}}.$ These data are included in the Additional file 1: Table S1.

## Discussion

This study aimed to determine whether impaired left ventricular filling or contractility (or both) were responsible for the attenuated cardiac reserve in people with uncomplicated type 2 diabetes. Our data confirmed that adults with type 2 diabetes had a smaller increase in cardiac output during steady-state semi-recumbent exercise caused by an attenuated increase in stroke volume. LV end-diastolic volume was smaller in T2D at rest and during semi-recumbent cycling at 40 and 60% of aerobic capacity, supporting an unproven contention that resting diastolic dysfunction, which is common in people with diabetes, reduces cardiac output during exercise. LV ejection fraction increased less during exercise in T2D and LV stroke work was lower in the T2D group, suggesting the T2D group also had impaired systolic function. However this clinical description of reduced systolic reserve, based on ejection fraction and LV stroke work did not reduce the magnitude or rate of LV emptying as end-systolic volumes and peak systolic shortening velocity (S′) were not different between the groups in any condition. Instead, these measures appear to be derived from the smaller end-diastolic volumes that persist in T2D during exercise. Based on these interpretations, we conclude that the reduced stroke volume reserve in our cohort with uncomplicated type 2 diabetes was an effect of decreased LV filling.

The novel finding of this study is the determination, using echocardiographic imaging, that diastolic filling of the left ventricle is reduced *during* semi-recumbent exercise. Impaired *resting* diastolic function, specifically reduced early diastolic filling velocity [2, 8, 14], is well-established in people with uncomplicated type 2 diabetes, and it has been implied that resting diastolic dysfunction is associated with reduced aerobic capacity in this cohort [14, 26]. However the loading conditions and diastolic duration of the resting ventricle do not reflect those during exercise, when late, or atrial diastolic filling has a greater contribution to end-diastolic volume [27, 28]. Indeed, Peterson et al. [15] found that resting early diastolic filling was inversely proportional to exercising stroke volume in healthy older people, suggesting that resting measurements of diastolic function have an arbitrary relationship with LV filling *during* exercise. In contrast, LV contractile responses to β-adrenergic stimulation are often [29–33] but not always [34] reduced in the diabetic heart, providing evidence that impaired systolic responses during exercise could limit cardiac reserve. In addition, a recent paper found that end diastolic volume responses to exercise were blunted, but end systolic volume responses were unchanged in adolescents with type 1 diabetes during supine exercise [35]. In this context, our finding that the T2D group achieved smaller end-diastolic volumes but almost identical end-systolic volumes to non-diabetic controls, supports the theory [14] that impaired diastolic filling limits aerobic capacity in people with type 2 diabetes but, at least in uncomplicated type 2 diabetes, systolic dysfunction does not.

It was beyond the scope of this study to determine the cause of reduced LV filling; nonetheless there are several factors which merit mention in the context of future study. LV compliance [36], early diastolic relaxation [2, 14] and total blood volume [37, 38] are all important to LV filling during exercise. Two studies (one in people with uncomplicated diabetes and another in type 1 diabetes) [8, 39] have found that total blood volume is reduced in people with diabetes, and that total blood volume is strongly correlated with LV end-diastolic volume. We found that mean total blood volume was 10% lower in T2D, however this was not significant (P = 0.09). We also found that total blood volume was only modestly correlated (r = 0.55; unpublished comparison) with end-diastolic volume. Our lack of statistical significance appears to reflect high variance in our sample; nonetheless we suggest that the modest association between these variables does not support our theory that total blood volumes affected left ventricular preload in this cohort.

The ratio of peak early to peak late mitral inflow velocity (E/A) was reduced and the peak velocity of the mitral annulus during early diastole (E′) was lower at rest in the T2D. Reduced velocity of early diastolic relaxation is well established in this cohort [2, 14] and would, in theory, cause a greater reduction in end-diastolic volumes as HR increases (i.e. diastolic filling time reduced). For example, Pinto et al. [3] found that the diminution of end-diastolic volume in adolescents with type 2 diabetes at rest became greater during submaximal exercise, suggesting the rate of diastolic relaxation did not ‘keep pace’ with a shortened diastolic filling time as HR increased. Under these conditions, we also found that end-diastolic volume was increasingly smaller in the T2D group as HR increased from rest (− 6.5%) to 40% $\dot{\text{{V}}}{\text{O}}_{{\text{2peak}}}$ (− 7.5%) to 60% $\dot{\text{{V}}}{\text{O}}_{{\text{2peak}}}$ (− 11.9%); however this interaction was not significant (P = 0.25). Technical limitations of Doppler echo prevented us from reliably quantifying mitral inflow velocities (unpublished data) during HRs above 100 bpm (where many of our exercise measurements were made). Similarly, repeated quantification of velocity–time integrals of mitral inflow waves had poor reliability in our lab. Therefore, we were not able to accurately determine peak flow rates during submaximal exercise and determine what contribution, if any this may have the smaller EDV among T2D.

The present study found that the HR of the two groups during their exercise stages were virtually identical (P > 0.05), therefore we would expect diastolic filling durations to be ‘matched’ during our exercise stages. An unexpected finding in this study however, was that diastolic duration was 5% lower in T2D, allowing less time for filling. Although this was not a focus of the study and did not reach significance (P = 0.06) Lalande et al. [8] also showed that T2D spent a smaller proportion of their cardiac cycle in diastole using cardiac MRI. Thus a non-significant reduction in diastolic filling time compounded by resting indicators of impaired relaxation may have contributed to the smaller EDV during exercise in T2D.

Finally, reduced LV compliance is an early cardiovascular manifestation of diabetes [4] which impacts LV filling [36]. Regensteiner et al. [16] suggested that increased stiffness limited LV filling during exercise in women with uncomplicated type 2 diabetes after finding that they had an exaggerated increase in LV end-diastolic pressure (pulmonary capillary wedge pressure) during incremental cycling exercise. However they used direct Fick and thermodilution methods to determine cardiac output, and were unable to confirm that these changes resulted in smaller LV volumes. We did not measure LV pressures, and can only assume that if our T2D cohort had greater LV stiffness, end-diastolic volume expansion would be reduced during exercise, when LV pre-load was increased. The T2D group had lower EDV across all conditions and increased EDV by 4.3% from upright rest to 60% $\dot{\text{{V}}}{\text{O}}_{{\text{2peak}}}.$ In contrast, the CON group increased their EDV by 10.6 at 60% $\dot{\text{{V}}}{\text{O}}_{{\text{2peak}}}.$ Despite these seemingly large differences in response, the change in EDV across conditions was not different between our groups (interaction P = 0.25). Therefore, while there is evidence that LV filling pressures rise more rapidly during exercise in people with diabetes, our data did not support the theory that these changes limited filling volume during exercise.

In conclusion people with uncomplicated type 2 diabetes did not achieve the same increase in cardiac output due to attenuated increases in stroke volume during exercise. Our data show that while LV systolic function did not appear to limit the T2D heart, people with T2D operated at smaller LV end-diastolic volumes throughout sub-maximal semi recumbent exercise. Therefore, impairments in LV filling appears to be an important contribution to the blunted cardiac reserve in people with diabetes during exercise.

## Additional file

**Additional file 1.** Means, standard deviation and statistical comparisons.
